# Management of Residual or Recurrent Disease Following Thermal Ablation of Renal Cortical Tumors

**DOI:** 10.15586/jkcvhl.2020.133

**Published:** 2020-06-09

**Authors:** Justin Loloi, W. Bruce Shingleton, Stephen Y. Nakada, Ronald J. Zagoria, Jaime Landman, Benjamin R. Lee, Surena F. Matin, Kamran Ahrar, Raymond J. Leveillee, Jeffrey A. Cadeddu, Jay D. Raman

**Affiliations:** 1 Division of Urology, Penn State Health Milton S. Hershey Medical Center, Hershey, PA, USA;; 2 Division of Urology, Augusta University, Augusta, GA, USA;; 3 Division of Urology, University of Wisconsin School of Medicine and Public Health, Madison, WI, USA;; 4 Department of Radiology and Biomedical Imaging, University of California, San Francisco, CA, USA;; 5 Department of Urology, University of California, Irvine Medical Center, Orange, CA, USA;; 6 Department of Urology, University of Arizona College of Medicine–Tucson, Tucson, AZ, USA;; 7 Department of Urology, The University of Texas MD Anderson Cancer Center, Houston, TX, USA;; 8 Department of Interventional Urology, The University of Texas MD Anderson Cancer Center, Houston, TX, USA;; 9 Bethesda Health Physician Group, Boynton Beach, FL, USA;; 10 Department of Urology, University of Texas Southwestern, Dallas, TX, USA

**Keywords:** cryoablation, radiofrequency ablation, renal cell carcinoma, nephrectomy, recurrences

## Abstract

Management of residual or recurrent disease following thermal ablation of renal cortical tumors includes surveillance, repeat ablation, or surgical extirpation. We present a multicenter experience with regard to the management of this clinical scenario. Prospectively maintained databases were reviewed to identify 1265 patients who underwent cryoablation (CA) or radiofrequency ablation (RFA) for enhancing renal masses. Disease persistence or recurrence was classified into one of the three categories: (i) residual disease in ablation zone; (ii) recurrence in the ipsilateral renal unit; and (iii) metastatic/extra-renal disease. Seventy seven patients (6.1%) had radiographic evidence of disease persistence or recurrence at a median interval of 13.7 months (range, 1–65 months) post-ablation. Distribution of disease included 47 patients with residual disease in ablation zone, 29 with ipsilateral renal unit recurrences (all in ablation zone), and one with metastatic disease. Fourteen patients (18%) elected for surveillance, and the remaining underwent salvage ablation (n = 50), partial nephrectomy (n = 5), or radical nephrectomy (n = 8). Salvage ablation was successful in 38/50 (76%) patients, with 12 failures managed by observation (3), tertiary ablation (6), and radical nephrectomy (3). At a median follow-up of 28 months, the actuarial cancer-specific survival and overall survival in this select cohort of patients was 94.8 and 89.6%, respectively.

## Introduction

The widespread use of abdominal cross-sectional imaging has contributed to the observed increased incidence and detection of small renal masses (SRMs) (1, 2). The majority of incidentally found SRMs are pathologically renal cell carcinomas (RCC) albeit with variable clinical behavior (3). Although the rates of RCC are increasing at 2–3% annually worldwide, most of these cancers are localized and accordingly are highly treatable with excellent disease-specific survival (4). The American Urologic Association (AUA) guidelines highlight the standard of care for the management of SRMs, including partial or radical nephrectomy when feasible, with surveillance and thermal ablation as appropriate in certain patient cohorts (5). Results of thermal ablation in appropriately selected patients can be comparable with surgical extirpation (6).

Incorporation of renal tumor ablation into clinical practice requires an understanding of the incidence and patterns of recurrence, which can occur in up to 20% of patients, depending on tumor characteristics (7). In general, if disease persistence or recurrence occurs, it typically manifests within the first few months following initial ablative therapy (8). Several options have been proposed for the management of a failed ablative procedure, including surveillance, salvage ablation, and partial and radical nephrectomies (9, 10). Salvage ablation, in particular for locally recurring RCC following cryoablation (CA), has been shown to be a feasible option with good outcomes and low complication rates (11).

When renal tumors are appropriately selected for thermal ablation, the likelihood of disease persistence or recurrence is low. Therefore, there is a relative paucity of information regarding specific management strategies to address residual or recurrent disease following renal thermal ablation. To this end, we reviewed a large multicenter database of patients with failed renal ablation to highlight subsequent management strategies.

## Patients and Methods

Following Institutional Review Board (IRB) approval at each of the participating institutions, a review of prospectively maintained institutional databases identified patients who underwent renal thermal ablation by either CA or radiofrequency ablation (RFA). Ablation was performed by either laparoscopic or percutaneous approaches, which were dependent on tumor size, configuration, and proximity to adjacent structures. Clinical records reviewed included office notes, operative reports, pathology and imaging data that identified those patients with oncologic recurrence following ablative therapy.

The primary outcome variables included evidence of renal oncologic recurrence and its subsequent management. Axial cross-sectional imaging studies with and without contrast were reviewed to identify disease recurrence. Specifically, the presence of contrast enhancement or lesion growth in the ipsilateral renal unit, including the ablation zone, was considered as evidence of radiographic local treatment failure. For those patients unable to receive contrast-based studies, evidence of residual or recurrent disease was confirmed by percutaneous renal mass biopsy (RMB).

Treatment failure was classified into one of three categories. Disease persistence or incomplete primary ablation was characterized by radiographic or biopsy evidence of viable carcinoma in the ablation zone on the initial radiographic study performed following the procedure (approximately ≤ 3 months). Recurrence in the ipsilateral renal unit was defined as evidence of carcinoma either in the ablation zone or within the ipsilateral kidney (by imaging or biopsy), following an initial negative imaging study. Metastatic or distant disease was characterized as evidence of RCC at a site outside of either renal unit.

## Results

Between August 2010 and March 2015, 1265 patients underwent ablation for renal cortical neoplasms at nine centers. Eight hundred and seventy five patients (69%) underwent RFA (683 percutaneous, 192 laparoscopic) and 390 patients (31%) underwent CA (183 laparoscopic, 207 percutaneous). Treatment approach was nonstandardized and was at the discretion of individual institutions and urologists. However, hilar tumors were generally managed laparoscopically to permit mobilization of the medial structures, including the colon, duodenum, and pancreas. Furthermore, the laparoscopic approach permitted identification of the hilar vessels, thereby minimizing the risk of procedural bleeding. All treatment approaches were performed collaboratively between urologists and interventional radiologists, with laparoscopic procedures completed by urologists and percutaneous procedures completed by both urologists and interventional radiologists.

Overall, 77 patients (6.1%) had evidence of disease persistence or recurrence at a mean interval of 13.7 months (range, 1–65 months), following ablation. Specifically, in those who underwent RFA, 6.6% experienced an oncologic recurrence (58/875), and in patients undergoing CA, 4.9% developed a recurrence (19/390). Of these, 21 were biopsied and all were positive for RCC. Table 1 highlights the characteristics of tumors with evidence of radiographic persistence or recurrence.

**Table 1:** Pre-treatment radiographic and pathologic characteristics of 77 renal tumors managed by ablation, with subsequent evidence of disease persistence or recurrence.

Size (cm; median, range)	3.1 (1.5–8.4)
Histologic subtype (No., %) Clear Cell Papillary Chromophobe Benign Nondiagnostic	51 (66) 13 (17) 2 (3) 3 (4) 8 (10)
Nuclear grade (No., %) * Low High Unspecified	23 (35) 32 (48) 11 (17)
Nephrometry score	6 (4 – 9)
Posterior (No., %)	46 (60)
Hilar (No., %)	6 (8)
Solitary kidney (No., %)	9 (12)
Biopsy confirmed recurrence (No., %)	21 (27)

* percentage based only on histology of cancer cases (n = 66).

Amongst those with ablation failure, 47 (61%) were classified as disease persistence (incomplete ablation) in the treatment zone; 29 (38%) as ipsilateral renal recurrence, all of which were in the primary ablation zone; and one (1%) with metastatic disease to the lung. Ipsilateral renal ablation zone recurrences were detected at a mean of 20.3 months (range, 4–59), following primary ablation. There were no differences in recurrence rates between percutaneous (56/890, 6.3%) and laparoscopic (21/375, 5.6%) approaches (P = 0.24). In addition, on further subgroup analysis, percutaneous RFA (49/683, 7.2%) had a higher recurrence rate, compared to percutaneous CA (7/207, 3.4%) (P = 0.05), but no other differences between any of the other subgroups, including laparoscopic RFA (9/192, 4.7%) and laparoscopic CA (12/183, 6.6%), were observed (P = 0.19).

**Figure 1:**
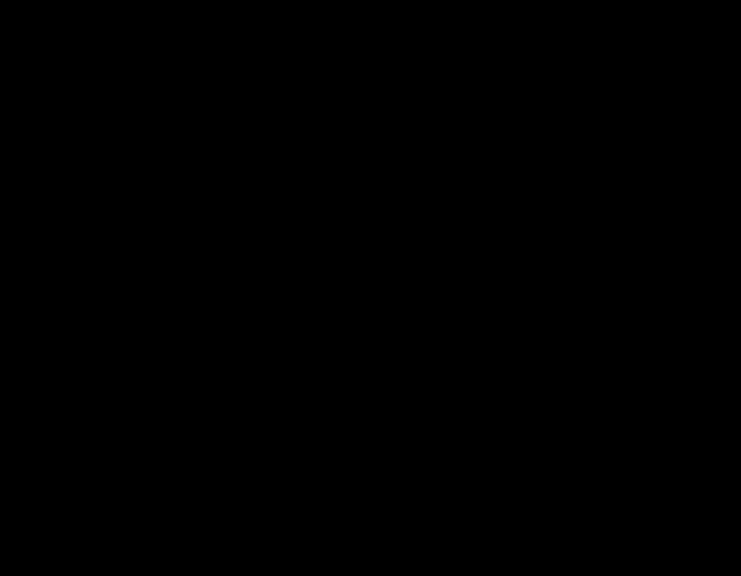
Management of disease persistence or recurrence following primary renal tumor ablation.

Figure 1 highlights the management strategies employed in those experiencing disease persistence or recurrence following RFA or CA. Of the 77 patients, 14 chose surveillance, 50 underwent secondary salvage ablation, and a total of 13 underwent extirpative surgery (five partial, eight radical). The patient experiencing metastasis had an 8.4-cm clearcell carcinoma in a solitary kidney with medical comorbidities. This patient developed pulmonary metastasis 7 months post-procedure managed by surveillance due to competing medical issues. In the cohort that proceeded with salvage ablation, a successful salvage procedure was documented in 38 patients, whereas 12 patients experienced recurrence after salvage ablation. In these 12 patients with a second postsalvage ablation recurrence, three underwent surveillance, six underwent a repeat tertiary salvage ablation, and three proceeded with radical nephrectomy. In the six patients who underwent tertiary salvage ablation, three experienced a successful procedure, whereas three developed a recurrence and subsequently elected for surveillance. The final pathology for the 16 patients who eventually underwent surgical extirpation was RCC in 11, necrosis/fibrosis in four, and oncocytoma in one.

Overall, at a median follow-up of 28 months, the cancerspecific survival and overall survival in this select cohort of patients with ablation failure was 94.8 and 89.6%, respectively.

## Discussion

The goal of this study was to use a large, multicenter renal thermal ablation dataset to specifically evaluate the rates of treatment failure and subsequent management strategies. Our observations noted that renal ablation carried out at high-volume centers demonstrates low treatment failure rates (~ 6%), with greater than 60% being attributable to incomplete primary ablation. Surveillance, salvage ablation, and surgical extirpation were all management options incorporated in practice with excellent cancer-specific survival and overall survival. These data are quite encouraging in presenting a framework for managing failures after renal ablative therapy, with enhanced patient counseling prior to the procedure.

Ablative therapies present a treatment option that can be successfully employed when managing a patient with an SRM (5). Indeed, early data from high-volume centers of excellence highlight favorable oncologic outcomes with a relatively low morbidity rate. In particular, Weld and colleagues highlighted in a summary of a published series that 4.6% of patients who underwent CA and 7.9% of patients who underwent RFA experienced a recurrence (12). Notably, our study, which represents some of the highest case numbers reported, demonstrated a similar recurrence rate of 6.1%, with only one patient experiencing a metastatic recurrence. The metastatic event was not entirely surprising given the challenging scenario of managing a cT2 renal mass in a medically comorbid patient with a solitary kidney.

Nonetheless, it is important to recognize that a percentage of patients undergoing renal ablation will invariably experience a treatment failure that requires subsequent management. In that regard, there is a relative paucity of literature delving into management options and outcomes following a failed renal ablation therapy. Breda et al. conducted a systematic review to identify renal ablation therapy cases and to provide an overview of treatment options in those experiencing recurrence. They demonstrated that for tumor recurrence following renal ablation, viable treatment options include active surveillance, repeat ablation, and salvage nephrectomy. Specifically, for patients with early enhancement (which may be attributed to postoperative inflammation), active surveillance of up to 1-year stands as a fairly reliable option and may prevent unnecessary therapy in those whose disease has been effectively managed (9). Similarly, Okhunov and colleagues demonstrated excellent results for salvage CA of T1a RCC in a smaller multicenter report (11). Our study similarly noted that while 18% of our patients elected for surveillance of suspected residual or recurrent disease, a similar proportion (20%) ultimately required extirpative salvage surgery.

Similar observations from Matin et al. noted that RFA patients had a 13.4% risk of residual or recurrent disease and CA patients had a 3.9% risk of treatment failure, following primary renal thermal ablation. These authors recorded a rather low therapy failure of 4.2% in those undergoing salvage ablative therapy, and for the population with recurrent disease, they documented an overall survival rate of 82.5% and a 2-year metastasis-free survival rate of 97.4% in those with localized, unilateral tumors. Akin to our study, a majority of recurrences were detected within the first 3 months following treatment (8).

It is important to recognize that ablation is most successful in the initial attempt and patients must be aware that salvage ablative procedures are (in general) likely to yield lower success rates. Indeed, in our series, the treatment failure rate of primary ablation was ~6%, secondary ablation was ~25%, and tertiary ablation was ~50%. Furthermore, our data suggest the potential value of RMB to better guide treatment in those patients with suspected recurrence. The absence of enhancement on contrast-based studies has been shown to be reliable in excluding viable carcinoma in ablated tumors (13). However, the presence of enhancement, particularly weak contrast uptake, is not pathognomonic for residual or recurrent disease and may represent inflammatory changes (14). Our results highlight that of those patients undergoing extirpative surgery, 25% had only necrosis or fibrosis on final pathology. In such patients, RMB may certainly guide *a priori* the subsequent need for therapy (15).

Newer techniques such as stereotactic ablative radiosurgery for renal cortical tumors may also certainly play a role in the primary or salvage treatments. A recent pooled analysis from the International Radiosurgery Oncology Consortium for Kidney (IROCK) underscored the importance of this treatment approach (16). Of 223 patients in this series, the rates of local control, cancer-specific survival, and progressionfree survival were 97.8, 95.7, and 77.4%, respectively, at 2 years; and they were 97.8, 91.9, and 65.4%, respectively, at 4 years. Such data highlight the promise of ablative options that are potentially even less invasive.

We acknowledge that this study is not without limitations. Our findings are limited by the study’s small sample size and its retrospective nature, and therefore treatment algorithms and strategies were at the discretion of individual clinicians. Furthermore, all tumors were not biopsied prior to management and therefore the treatment success and failure rates were based on treating enhancing SRMs some of which may not be pathologically kidney cancer. In addition, pathologic information and radiographic characteristics of the entire cohort of treated patients (n = 1265) were not available to determine factors predictive for disease recurrence. Nonetheless, we believe our data offer large-scale insight into specific patterns of treatment and outcomes in those with residual or recurring disease following renal ablation.

## Conclusions

Oncologic recurrence is an important consideration in patients undergoing ablation therapy for renal masses. For those experiencing a recurrence, there are several management strategies that can be employed for disease control. Careful delineation of algorithms for residual or recurring disease permits optimized patient counseling prior to primary ablation therapy. Experience and lesion selection may play an important role in guiding successful ablation.
